# Randomness in the Bedroom: There Is No Evidence for Fertility Control in Pre-Industrial England

**DOI:** 10.1007/s13524-019-00786-2

**Published:** 2019-06-17

**Authors:** Gregory Clark, Neil Cummins

**Affiliations:** 1 Department of Economics, University of California, Davis, Davis, CA USA; 2 Department of Economic History, London School of Economics, London, UK; 3 Centre for Economic Policy Research (CEPR), London, UK

**Keywords:** Pre-transitional fertility, Natural fertility, Spacing, Birth intervals, Parity-specific birth control

## Abstract

Overturning a generation of research, Cinnirella et al. *Demography, 54,* 413–436 (2017) found strong parity-dependent fertility control in pre-Industrial England 1540–1850. We show that their result is an unfortunate artifact of their statistical method, relying on mother fixed effects, which contradicts basic biological possibilities for fecundity. These impossible parity effects also appear with simulated fertility data that by design have no parity control. We conclude that estimating parity control using mother fixed effects is in no way feasible. We also show, using the Cambridge Group data that Cinnirella et al. used, that there is no sign of parity-dependent fertility control in English marriages before 1850.

**Electronic supplementary material:**

The online version of this article (10.1007/s13524-019-00786-2) contains supplementary material, which is available to authorized users.

## Introduction

Historical demography has failed to find substantial evidence for parity-dependent birth control before the demographic transition. Hence, pre-industrial humans could be thought of as exhibiting natural fertility (Henry 1961). Wrigley et al. (1997:461) concluded that “early modern English parishes were communities in which ‘natural fertility’ was the norm.” Recently, however, Cinnirella, Klemp, and Weisdorf (hereafter, CKW) discovered strong parity-dependent fertility limitation in pre-Industrial England all across the interval 1540–1850 (Cinnirella et al. 2017). Using the Cambridge Group’s *family reconstitution* data for 15,000 families and 71,000 births, Cinnirella et al. used Cox proportional hazard models to estimate the hazard of a birth.^1^ Their crucial empirical innovation was to include mother-specific fixed effects (or stratified hazards) in the estimation to control for fecundity differences across couples.

CKW’s table 4 gives the key results. Before they introduced household-level stratification/mother-level fixed effects to the Cox proportional hazard model, they found no parity effects for any of parities 2–6 compared with parity 1; parity here is defined as net parity, the number of surviving children at the start of each interval. After they introduced individual fixed effects, however, they found very strong parity-dependent control. For example, the hazard of birth at net parity 6 and above was only 6 % as large as that of parity 1. This is an extraordinarily powerful result and is illustrated neatly by their figure 2.

To back up this estimate, CKW also estimated a maternal age effect by further performing their analysis using mothers only within 5- or 10-year age groups (Cinnirella et al. 2017: table 5).^2^ Even within narrow age groups with no age effects, mother fixed effects reveal strong and substantial parity-dependent declines in birth hazards. It would appear that CKW have uncovered the holy grail that eluded scholars before them: substantial parity-dependent fertility control long before the demographic transition and long before the Industrial Revolution.

We demonstrate, however, that the CKW parity effects are entirely an artifact of the specifications they chose. When estimated for mothers over the whole course of their marriages, the parity estimates imply biologically impossible levels of fecundity at higher ages. When estimated for the truncated 5- to 10-year intervals, CKW predict fertility levels more than three times smaller than those observed, in the out of sample years of marriage. When factors exogenous to mother fecundity, such as changes in age at marriage, change net parity at age 30, the birth rate of mothers aged 30–34 is similar across all net parities despite substantial parity differences among these mothers.

Remarkably, the spurious parity effects are generated in different ways in CKW’s tables 4 and 5. In CKW’s table 4, the spurious parity effects appear after mother fixed effects are introduced because in that case, the regression coefficients are attempting to model for each mother a sequence of births followed by spacings of typically two to four years. In their method, changes in net parity coincide with a birth. On average, parity is increasing with births. Thus, an increase in net parity is associated with an immediate decline in the probability of a birth followed by an increase. The regression predicts this pattern best by assuming a strong positive age effect in fertility and an ever-stronger negative parity effect at higher parities. But these odd regression coefficients, appearing within a constrained model, have no causal interpretation.

In CKW’s table 5, the spurious parity effects are created by an entirely different mechanism: the censoring created by using a 5- to 10-year window. With mother fixed effects, a mother contributes to the estimated parity effect with the CKW specification only if she has two births in the age window, thus producing a change in net parity. The first birth in the interval, when parity is lower, will be followed by a second birth in the interval only when the first birth interval is less than 5 years. Because that first birth can occur any time in the interval, the censoring of the first birth interval will be even stronger. The second birth interval, when parity is typically higher by one, is uncensored. In fact, the Cambridge Group data contain no evidence that birth rates were parity-dependent in English families for marriages before 1850, as we will show in this comment. The traditional view of an absence of any evidence of birth control through spacing is vindicated.

## Why the CKW Results Cannot Be Correct

**Fig. 1:**
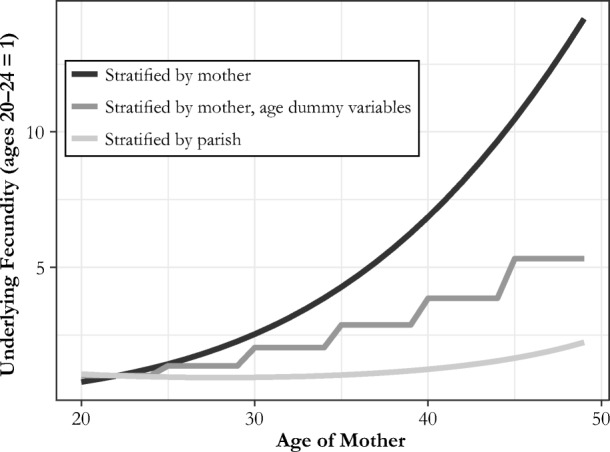
CKW estimated age effects on fecundity, with and without mother-level stratification

A number of tests show that CKW’s estimated parity effects are an artifact of the specification and cannot possibly represent actual causal behavior. The first test is based on CKW’s table 4. CKW controlled for age effects in fecundity using a quadratic specification. The effect of age on underlying fecundity is not immediately evident from their table 4. Figure 1 in this comment shows the implied age effect on fecundity in their estimation, with and without mother fixed effects. The age effects are such that implied fecundity is seven times as great at age 40 as at ages 20–24, a clear biological impossibility. This result does not just represent a failure of the fit at the extremes of age. Fecundity at ages 30–34 is estimated to be nearly three times that at ages 20–24. In contrast, when only parish fixed effects are employed, fecundity is flat within ages 20–40.^3^ The figure also shows the age effects when we employ a more conventional strategy of indicators for ages 15–19, 20–24, 25–29, 30–34, 35–39, and 40–44. These effects are not as extreme, but they still show an impossible substantial upward path for underlying fecundity with age.

These impossible age effects are not only an idiosyncrasy of the regression fit but a necessary consequence of the strong estimated parity effects. Because women in their 30s at parity 6 are still having a child every two to three years, their implied fertility levels at parity 1 in their teens or 20s would be more than 30 times as great if fecundity followed the standard pattern with age. They would be having a child every month or two. To fit the data, fecundity at earlier ages must be substantially lower. The parity effects are thus an artifact of the bizarre fit of the age controls to the data.

**Fig. 2:**
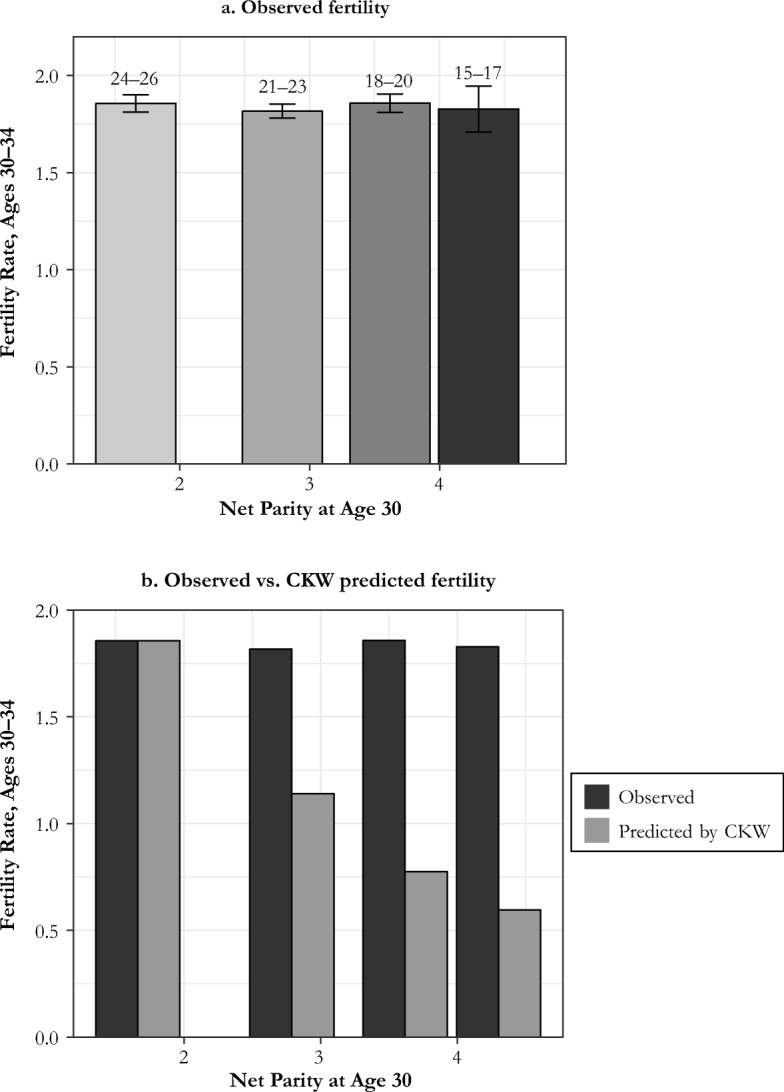
Observed and implied birth rates, 30–34, by age at marriage.

A second way to see that CKW’s parity results are an artifact is to consider their estimates from the short panel regressions reported in their table 5. CKW’s raw data show, for example, that the fertility rate increases in net parity at age 30 for ages 30–34. Their table 5 estimate suggests, however, that women of net parity 4 have fertility rates that are only 5.5 % of those they would experience at parity 1. CKW argued that these raw data are compatible with parity control because the higher-parity women have intrinsically higher fecundity rates. But the estimated hazards imply that if some circumstance caused these women to be brought back to parity 1 at age 30, they would then experience an impossibly high birth rate: a childbirth every two months. Figure 2 in this comment shows the actual birth rates of women who have parities 1, 2, 3, and 4 at age 30 over the years 30–34, and the birth rates that would result, based on the estimates, if they were reduced to parity 1. Again, these are biological impossibilities. According to these estimates, the parity 4 group would have had more than six births per year at parity 1. Whatever the estimates from CKW’s tables 4 and 5 show, they do not describe actual fertility control but rather an artifact of CKW’s statistical models.

In addition, when differences in net parity at age 30 are generated by a factor not strongly linked to underlying fecundity—age at marriage—the estimated effects from CKW’s version of the Cox model fail to appear. Figure 2, for example, looks at the fertility of women aged 30–34 in the family reconstitution study as a function of their age at marriage. Age at marriage is a strong predictor of net fertility at age 30. Those marrying at ages 15–17 had, on average, 4.30 surviving children at age 30; the comparable figure for those marrying at ages 24–26 was 1.66. Yet, as panel a of Fig. 2 shows, birth rates at ages 30–34 did not differ across ages at marriage. If we apply to these data CKW’s estimate of parity effects and take those marrying at ages 24–26 as the reference group, then implied birth rates for those marrying at ages 15–17 are one-third of the level observed. Panel b of Fig. 2 shows both the observed and the CKW-estimated birth rates at ages 30–34 by age at marriage.

Actual and predicted fertility could be made to fit for those with different marriage ages only if women marrying earlier had three times the levels of fecundity of women marrying late. But this relative fertility rate cannot be reconciled with the fact that net parity at age 30 increased nearly linearly with earlier ages of marriage. If the youngest brides were so much more fecund than those marrying later, they would show much higher net parity at age 30 than was the case. The CKW-estimated parity effects simply do not exist in reality.

## The Generality of CKW’s Results

CKW’s results are not just a product of the Cox proportional hazards model. Similar results would appear of seeming substantial parity control if mother fixed effects were applied to pre-industrial English fertility data using either an OLS specification or a logistic specification. Thus, the odd effects CKW found are of a very general nature. Further, these results can be generated in a simulated data set of English fertility pre-1850 in which, by design, parity control is completely absent.

We create a simulation of fertility designed to match the characteristics of marriage in England in the Wrigley et al. (1997) data for 1800–1837 in terms of the average age at first marriage, variance in age at first marriage, and the age pattern of fertility. A central feature, however, is that each woman is assigned a randomly generated fecundity fixed effect of mean 1 and standard deviation 0.3 (denoted *F*^0^) to allow significant mother fixed effects. In this simulation, fertility rates are independent of parity by construction. Women marry and experience potential fertility until age 50. First marriage age for women is assigned as a random variable, with a mean and variance specified to match the family reconstitution data.

For each marriage year, a woman generates a fertility probability (*Prob*(*B*)) for a given age *t* according to Eq. (1):

1 $$ Prob{(B)}_t={F}^0\bullet {F}_t^{Observed}, $$

where ${F}_t^{Observed}$ are the age-specific fertility rates of the family reconstitution sample. Gross parity is calculated as the integer value of the cumulative sum of the age-specific birth probabilities. To this, we apply a child mortality effect. Each birth receives a random number between 0 and 1. A value above .4 indicates that the child lived; a value below .4 indicates that the child died in its first year. This child mortality effect allows us to estimate the hazard model by net parity.^4^

In Table 1, we apply the CKW spacing model to these simulated data. We find a rising hazard of a birth with parity. This finding simply reflects the mechanical fact that women who are more fertile are more likely to have a birth.

**Table 1:** Simulation of random fecundity, with no parity control by construction and with net parity

	Hazard of a Birth			
	(1)	Stratified	(3)	Stratified
		(2)		(4)
Age	1.309**	2.596**		
	(0.021)	(0.086)		
Age (squared)	0.994**	0.987**		
	(0.0002)	(0.0004)		
25–29			0.783**	2.195**
			(0.029)	(0.107)
30–34			0.548**	3.858**
			(0.022)	(0.274)
35–39			0.368**	4.826**
			(0.016)	(0.433)
40–44			0.153**	2.887**
			(0.009)	(0.302)
45–49			0.021**	0.439**
			(0.002)	(0.063)
Net Parity 2	1.046**	0.349**	1.038*	0.423**
	(0.018)	(0.013)	(0.018)	(0.012)
Net Parity 3	1.070**	0.139**	1.044^†^	0.199**
	(0.024)	(0.009)	(0.024)	(0.009)
Net Parity 4	1.105**	0.063**	1.044	0.102**
	(0.032)	(0.006)	(0.031)	(0.007)
Net Parity 5	1.087*	0.030**	1.008	0.054**
	(0.045)	(0.004)	(0.044)	(0.005)
Net Parity 6+	0.995	0.008**	0.922	0.019**
	(0.049)	(0.001)	(0.047)	(0.002)
Number of Observations	33,483	33,483	33,442	33,442

*Notes:* The table presents exponentiated coefficients from a Cox model.

^†^*p <* .10; *p < .05; **p < .01

**Fig. 3:**
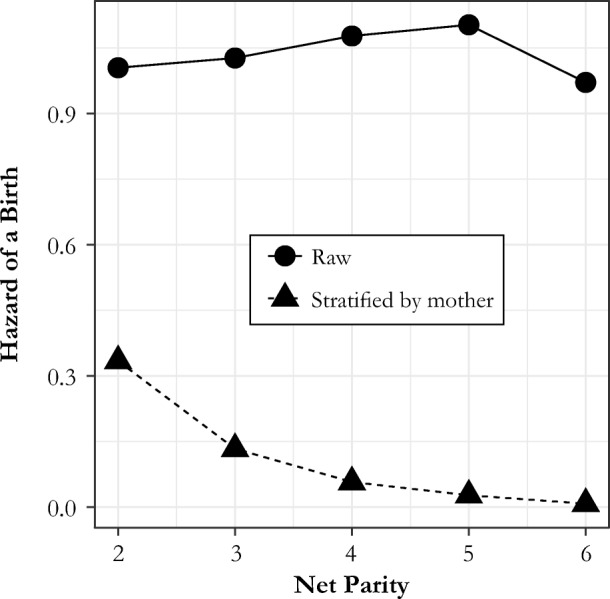
Spurious parity hazards from simulated data. Relative hazards from Cox models on simulated data with no parity control by construction.

When we apply mother level stratification in columns 2 and 4, however, we find the same pattern that CKW found in the Cambridge data. It appears that the simulated women are controlling their fertility. The hazard of a birth is 15 to 20 times lower at parity 5, which we know to be impossible given the construction of the data. This pattern can only be an artifact of the empirical model. Figure 3 illustrates the sudden emergence of this illusory parity control after mother stratification is introduced to the Cox model.

Our Table 2 replicates the Cox model for parity by age group. Again, we find that our simulated mothers exhibit parity control even though we know this is impossible by construction. Clearly, something has gone very wrong in the estimation. In our simulation, we can extract the programmed fecundity fixed effects (*F*^0^) and compare them with those estimated by the Cox model. Fig. A2 in our online appendix reveals that underlying fecundity (*F*^0^) is badly misestimated by the mother fixed-effects empirical model for all age groups.

**Table 2:** Simulation of random fecundity, with no parity control by construction and with survey time as dependent variable

	20–24	25–29	30–34	35–39	40–44	45–49
	(1)	(2)	(3)	(4)	(5)	(6)
Net Parity 2	0.513**	0.421**	0.201**	0.063**	0.000**	0.000**
	(0.033)	(0.017)	(0.012)	(0.009)	(0.000)	(0.000)
Net Parity 3	0.318**	0.187**	0.047**	0.004**	0.000**	0.000**
	(0.052)	(0.013)	(0.004)	(0.001)	(0.000)	(0.000)
Net Parity 4+	0.225**	0.079**	0.011**	0.0003**	0.000**	0.000**
	(0.044)	(0.008)	(0.001)	(0.0001)	(0.000)	(0.000)
Number of Observations	1,132	4,318	6,434	7,063	7,235	7,260

*Notes:* The table presents exponentiated coefficients from a Cox model with mother-level stratification.

**p < .01

In addition, with the simulated parity control–independent data, if mother fixed effects are allowed in an OLS or logistic specification, the spurious parity control effects again appear. Thus, mother fixed effects create mischief across a wide range of model specifications.

## What Creates the Fixed-Effects Artifact

Why do mother fixed effects in CKW’s table 4 produce the impossible net parity effects? Our interpretation is that the switch to mother fixed effects dramatically changes the pattern that the regression coefficients attempt to capture. Without mother fixed effects, the predicted hazard of a birth in any year is a smooth function of age. At any mother age, a variety of mothers of a given net parity previously gave birth in any one of a number of years.

**Fig. 4:**
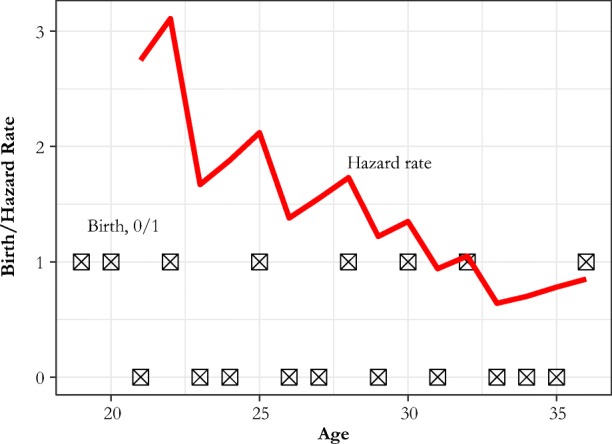
The fertility history of Margaret Ellmers. 0 is a year without a birth, and 1 is a year when a birth is recorded. The red line is the implied CKW hazard rate.

However, with mother fixed effects, the regression attempts to fit for each mother a pattern of births to a hazard function. Take, for example, the case of Margaret Ellmers, who married in 1824 at age 21 and gave birth to eight children.^5^ These children were born at mother’s age 21, 23, 26, 29, 31, 33, 37, and 42, with no child deaths before age 42. Our Fig. 4 shows the pattern of 0s (years without a birth) and 1s (a year with a birth) that correspond to Margaret’s fertility history and CKW’s estimated hazard ratio from their table 4 (with arbitrary scale) for Margaret. The combination of net parity effects (negative) and age effects (positive) creates a *sawtooth* decline in the hazard of a birth with age. The substantial parity effects, which typically increase immediately after a birth, allow the hazard of birth to decline after each birth before rising again through the substantial age effects. This is also why the hazard of birth declines by about the same proportion for parities 2, 3, 4, 5, and 6+.

Because of the individual mother fixed effects, which can be at any level, this pattern of hazards can be fitted with the parity effects to mothers who marry at any age. CKW tried to control for the infertile period following a birth by beginning the next birth interval nine months after a birth. Breastfeeding, however, leads to an interval of depressed fertility that is longer than nine months, so the effect still appears. The failure here is that in the constrained model, what fits best in a statistical sense does not imply what fits best in causal terms. It may be that we can predict the outcomes best with a mechanism of rapidly rising fertility offset by strong negative parity effects. But that does not imply that mothers are using net parity in any sense to inform birth decisions.

The mechanism of the regression fit is illustrated here by what happens when we switch to five-year age indicators. This fits the pattern of rising hazards after a birth only if the interval to the next birth crosses a five-year age period. Thus, the parity effects found with the five-year age indicators are weaker, and the rise in estimated underlying fecundity with age substantially less.

CKW did not include age dummy variables when they made their short panel estimates in their table 5. When we add these dummy variables, we see two results. First, the age dummy variables show sharply rising fecundity—impossible increases in fecundity (as in CKW’s table 4)—even over the five-year interval. Second, when the age dummy variables are added, the parity estimates become stronger.

CKW’s table 4 could be estimated without age controls, but because net parity is strongly correlated with age, any effect of age on fecundity would be mistakenly picked up as a parity control effect. One might even consider applying a forced fecundity adjustment with age to the data. But if one suspects active parity control, then what would that adjustment be? Any observed age effects on fecundity would be a mixture of pure age effects and parity control. If the age effect is in any way incorrect, then some sort of parity control would be estimated. There seems no way to correct the specification in CKW’s table 4 to produce a reliable causal parity effect estimate.

Why then does the estimation in CKW’s table 5, with no age controls, produce similar substantial parity effects? The reason is that CKW censored the birth intervals at lower parities in these estimations.

CKW’s table 5 presents estimates from the Cox model with complete birth intervals by 5- to 10-year mother age periods. Consider the estimate for ages 30–34. A mother contributes to the estimate of fertility at net parity only in the following circumstances. She has a first birth at ages 30–34, which generates the beginning of the first interval at net parity *N*. She has a second birth before age 35, which generates the end of the first interval and beginning of the second interval at parity *N* + 1. The end of the second interval can be a birth at any age. For CKW’s mother ID 15, for example, the first birth was at age 30.3, the second was at age 34.5, and the concluding birth was at age 42. For most mothers, just two births will be observed at ages 30–34. Thus, the birth interval is truncated at *N* but not at *N* + 1. The birth interval at *N* has to be less than five years in order for the observation also to have a birth interval at *N* + 1. But because the first birth at ages 30–34 will often be at age 31 or 32, the length of the birth interval at parity *N* can be truncated to as little as one year.

With mother fixed effects, this censoring will generate a spurious estimate of steadily increasing birth intervals for parities 1, 2, . . . *N*. At younger ages, an additional element in the censoring of the earlier parity interval is that women may not marry until well into the interval, leaving very small amounts of time for the required two births. For example, most women will marry later in the age interval 15–24.

**Fig. 5:**
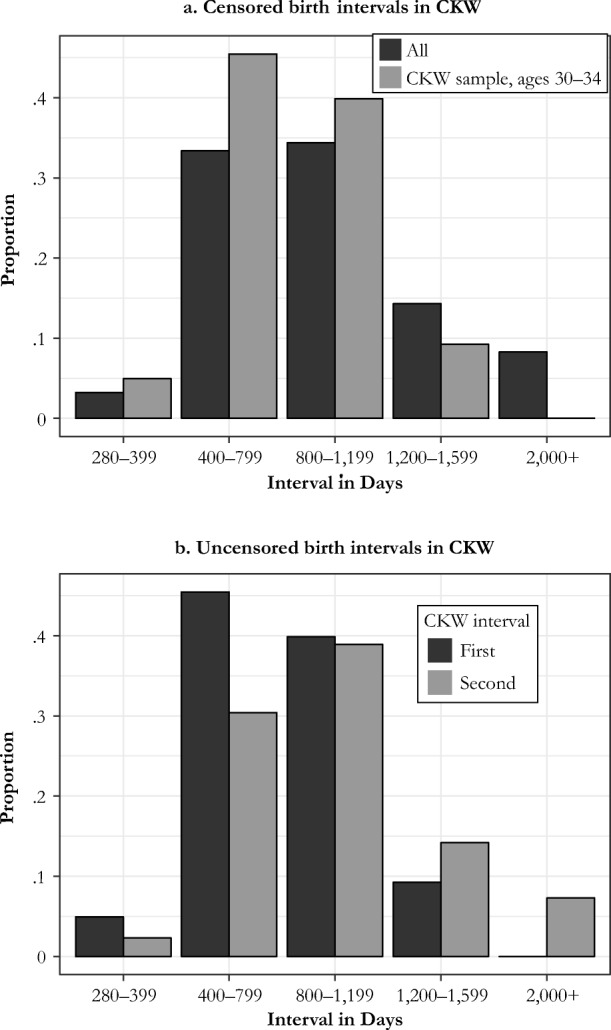
Censoring in CKW’s analysis

Panel a of Fig. 5 shows an example of the effects of this censoring in the CKW family reconstitution sample. The distribution of the interval after the first birth at ages 30–34 at net parity *N*, in days, is shown as the black column. The gray columns show the interval for births included in the sample aged 30–34, where the second birth also falls at the mother ages 30–34, so that the data contribute to the estimate. The average of all birth intervals following the first birth after age 30 is 1,079 days. The average for the births that contribute information to the estimation in the sample aged 30–34 is 822 days—almost 25 % shorter.

The second birth interval, at parity *N* + 1, is not censored in their design. Thus, the distribution of this second interval is correspondingly longer than that of the first, as shown in panel b of our Fig. 5. The average length of the second birth interval observed at ages 30–34 is 1,087 days.

We can easily demonstrate the importance of this censoring in generating the effects shown in CKW’s table 5. For the mother age group 30–34, CKW’s table 4 shows their estimates, estimates for a shorter window of 30–33 years, and estimates for a longer window of 30–38 years.^6^ As can be seen in Table A2 of our online appendix, the shorter the window, the more the censoring and the more pronounced CKW’s parity effects. For somewhat arbitrary reasons, CKW considered only mothers with final parities of 5 or lower in their table 5 estimates, despite using parities up to 6 or more in their table 4. It is a pity they made that restriction given that they had plenty of data to estimate net parity effects up to parity 8 and above. Table A2 in our online appendix shows these estimates for ages 30–34. Now it becomes more evident that the parity effects are an artifact. The estimated hazard rates at parities 8 or more drop to 1.5 % of the rate at parity 1, implying that no woman would ever reach a parity 9 without having fecundities many times the average. In reality, plenty of women reached parity 9 and above.

It might seem that we could get a correct estimate for ages 30–34 using an unrestricted panel of those aged 30 and older. However, we would then be lengthening the time intervals between party *N* and parity *N* + 1, with all the accompanying biases in the fixed-effects estimation. If we estimate the four-year CKW panel of women aged 30–34, including time dummy variables for mother aged 31, 32, and 33–34.0, then the time effects are dramatically positive with age and are statistically significant. The net parity effects also become much stronger.

There again seems no way to redeem the short panel estimates. A short panel will induce wild biases. A long panel gets us back to the age effect controls of CKW’s table 4, which then present insurmountable problems.

## No Evidence of Fertility Control in England Before 1850

The preceding material shows that CKW’s estimates of significant parity effects are an artifact. Here we consider whether any case can be made more generally to overturn the long-standing consensus that there is no evidence of conscious control of fertility within marriage in pre-industrial England (Wilson 1984; Wilson and Woods 1991; Woods 2000; Wrigley et al. 1997).

The rest of CKW’s article shows connections between birth intervals and variations in wage rates as well as occupational status (Cinnirella et al. 2017: table 3). But such connections do not in themselves imply that men and women in pre-industrial England were controlling family size within marriage. If birth spacing was higher for some occupational groups, that could well be just a product of the location and lifestyles of that group. If birth spacing lengthened when real wages were lower, that could be just a product of nutritional stress. For example, in separate research (Clark and Cummins 2015) we found that birth intervals for poorer families were, on average, longer than for richer ones for marriages in England before 1780. Does this finding demonstrate early fertility control by poorer families or instead the effects of unmeasured life circumstances on fecundity?

The one obvious mechanism to control family size in pre-industrial England was delay in the wife’s age at marriage. CKW’s table 3 shows no significant connection between variation in real wages across periods and women’s age at marriage. They found some modest occupational associations with age at marriage and occupational categories, but these associations could be due to the lifestyle and environment associated with certain occupations.

In this section, we use the Cambridge Group data to examine whether there is any sign of parity control in pre-Industrial England. We look at two tests.^7^

1. Does early age at marriage, which predicts greater net parity at later ages, reduce fertility at ages 30–34?

**Fig. 6:**
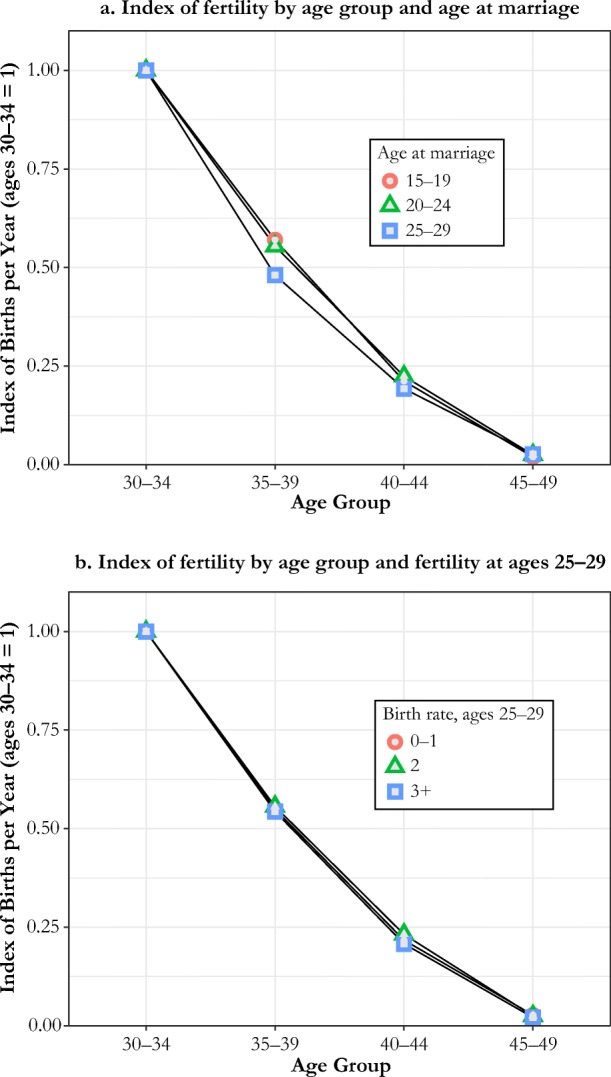
Simple tests of parity control by variations in parity, from age at marriage and observed birth rates: Cambridge Group data

We showed in Fig. 2 using the Cambridge Group data that parity differences caused by differences in mother’s age at marriage have no effect on the birth rate at ages 30–34. In the Cambridge Group data, at age 30, women who married at ages 15–19 had a net parity of 2.95, women who married at ages 20–24 had a net parity of 2.33, and women marrying at ages 25–29 had a net parity of 1.41. Panel a of Fig. 6 shows the decline in fertility rates from ages 30–34 to 35–39, 40–44, and 45–49 for these marriage groups. The decline is proportionately exactly the same, as would be consistent with absence of parity-dependent control.

2. Does fecundity at ages 25–29 influence the decline in birth rates at ages 30–44?

CKW’s central argument was that accounting for individual fecundity resulted in the discovery of parity control. Couples undoubtedly vary by fecundity. Here, again using the Cambridge Group data, we divide women who married in the same interval (at ages 20–24) into three fecundity classes based on their number of births in the early years of marriage at ages 25–29: 0–1, 2, and 3 or more. These women had substantial differences in net parity by age 30: 2.1, 3.0, and 3.6. If there was fertility control based on net parity, then those with higher fecundities in the early years of marriage should see more of a decline in fertility later. We take their fertility rates at ages 30–34 as the baseline.^8^ As panel b of Fig. 6 shows, the relative fertility decline at ages 35–39, 40–44, and 45–49 compared with 30–34 was exactly the same across the three groups of women, despite their continuing differences in net parity. This finding is consistent with the hypothesis that differences in fecundity were biologically determined, and these differences continued to be relatively unchanged across the course of marriages despite the differences they created in net parity over time. It is clear that unobserved heterogeneity in fecundity across couples is not masking fertility control in pre-Industrial England.

Of course, if there was fertility control throughout marriage, then the lower birth rate for women ages 25–29 could already have been controlling fertility. But this would imply that fertility control in pre-industrial England took the form of longer spacing throughout marriage rather than earlier stopping. Thus, this test rules out stopping as a mechanism of fertility control in pre-industrial England.

## Conclusion

CKW’s introduction of mother fixed effects into the estimation of the influence of net parity on birth hazard rates seemed a promising and productive research innovation. However, their discovery of parity-dependent fertility limitation in pre-Industrial England across the interval 1540–1850 is, unfortunately, an artifact of these fixed effects. When the estimate is made for the lifetime of mothers, a statistical fit emerges that does show huge negative parity effects. However, this is a predictive fit only: it makes no sense as a causal interpretation of the behavior of couples. This is revealed when we consider the substantial rise in fertility with age that is implied by this fit; when we look at the birth rates of mothers who have different net fertilities at age 30 because of different ages of marriage and find no decline in birth rates associated with higher parities; and when we construct simulated fertility data that by construction have no parity effects and find CKW’s strong parity effects along with systematic misfitting of the assigned differences in mother fecundity.

CKW’s estimates of parity effects using short mother age panels with mother fixed effects also create spurious parity effects, this time because of censoring of lower-parity birth intervals. We can see no way to introduce mother fixed effects into parity control estimations without inducing these spurious associations. Because net parity is associated with age, age has to be controlled for, and attempting to do so always induces spurious parity effects as well as biologically implausible or impossible age effects.

Indeed, CKW presented no clear evidence of any deliberate fertility control of any kind in pre-Industrial England. All the other associations they found between real wages, occupations, and age at marriage and birth spacing could be potentially explained as simply the product of nutrition, living environments, and social patterns rather than deliberate fertility control.^9^

Finally, using the Cambridge Group family reconstitution data, we find no sign of any parity control in marriages before 1880. Net parity differences induced by differences in age at marriage had no influence on birth rates at ages 25–29 or 30–34. Birth rates did not decline more quickly after age 35 for women who married earlier and thus had higher parities later. Women who were more fecund at ages 25–29 and who thus had higher parities by age 30 did not show any greater decline in fertility between 1830–1834 and 1835–1839 or 1840–1844. The traditional view of an absence of conscious birth control in pre-industrial England appears to be correct.

For all the reasons noted in this conclusion, we are confident that any attempt to establish parity control using family fixed effects will succeed only when the estimation involves mothers’ fecundity increasing (or nondeclining) with age and that the stronger the estimated effects, the more biologically impossible the mothers’ increasing fecundity with age will be. We invite interested readers to check for themselves the implied age-fecundity effects in the tables in the authors’ response to this comment.

## Electronic supplementary material

ESM 1 (PDF 191 kb)

## Footnotes

1 See Wrigley et al. (1997) for a complete description of the data.

2 CKW’s code contains a minor programming error such that the age groups were in fact just 4 and 9 years, but this did not substantially affect any results. We discuss this further later in this comment.

3 The age effects on fecundity in this case still do not comport well with the underlying biological realities. Controlling for age effects is thus much better accomplished with age indicators than with a quadratic expression that imposes constraints on the fit.

4 In our online appendix, Fig. A1 reports the accuracy of the model in estimating fertility by age and by aggregate distribution; Table A1 reports the summary statistics for this more complex simulation.

5 Margaret Ellmers’s fertility history was reconstructed using parish registers, census records, and family genealogies for the study Families of England (http://www.familiesofengland.com/).

6 CKW stated that they estimated the panel effects shown in their table 5 using five- and ten-year windows. In this case, the age interval 30–34 would be 30–34.99. In fact, their Stata code has an error such that the actual intervals in their table 5 are four and nine years (15–24.0, 25–29.0, 30–34.0, and 35–44.0), with almost a year missing between each panel. This will only increase the censoring of the earlier parities.

7 Using an alternative fertility database, we also conducted a third test based on the sex composition of surviving children at age 30. We found no evidence of gender-related parity control.

8 If we use 25–29 as the baseline, then we will observe spurious regression to the mean because there is randomness in births and because we used the 25–29 period to group the mothers.

9 CKW included temperature and the crude death rate as controls in their analysis, which only imperfectly controls for nutritional stress, living environments, and social patterns. Outside England, scholars have found evidence of fertility control in response to short-term economic stress but not parity-specific control. For example, Bengtsson and Dribe (2006:742) found that food prices and the timing of births were correlated in Southern Sweden in 1766–1864 but that the population “showed no signs of parity-specific control.” Also, Dribe and Scalone (2010:411) found “deliberate, but non-parity specific, control” by analyzing the correlation of grain prices and births in six German villages in 1766–1863. In Europe before 1850, France is the only country with clear evidence for widespread parity-specific control, which originated among the rich (Cummins 2013).
