# Supplementary material for: Randomness in the Bedroom: There Is No Evidence for Fertility Control in Pre-Industrial England
Source: Demography. 2019 Jun 17;56(4):1541–55. doi: 10.1007/s13524-019-00786-2 (PMC6667415; doi:10.1007/s13524-019-00786-2)
Supplement: Supplementary file 1 — (PDF 191 kb) [file 13524_2019_786_MOESM1_ESM.pdf]

# Appendix [Online]

## A Extra Tables and Figures

**Table A1** Summary Statistics, Complex Simulation Data

| Statistic | N | Mean | St. Dev. | Min | Max |
|---|---|---|---|---|---|
| Age | 40,545 | 35.86 | 8.00 | 20 | 49 |
| Fecundity | 40,545 | 1.00 | .30 | .10 | 1.94 |
| Birth | 40,545 | .21 | .41 | 0 | 1 |
| Child Death | 40,545 | .08 | .28 | 0 | 1 |
| Parity | 40,545 | 3.97 | 2.75 | 0 | 19 |
| Net Parity | 40,545 | 2.36 | 1.90 | 0 | 16 |

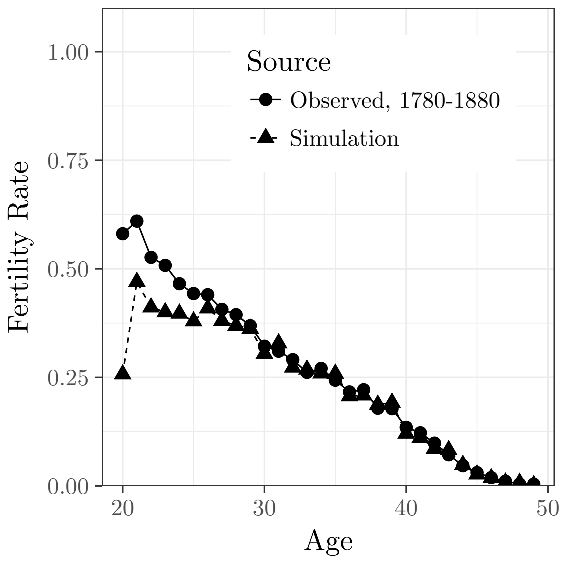


**(a)** Age Specific Fertility

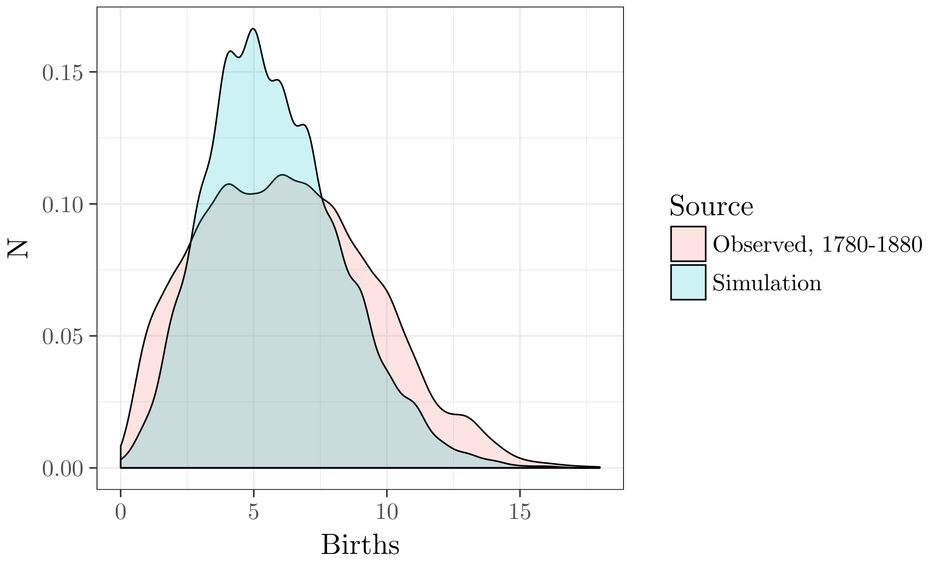


**(b)** Distribution of Births

**Figure A1** Simulation Calibration

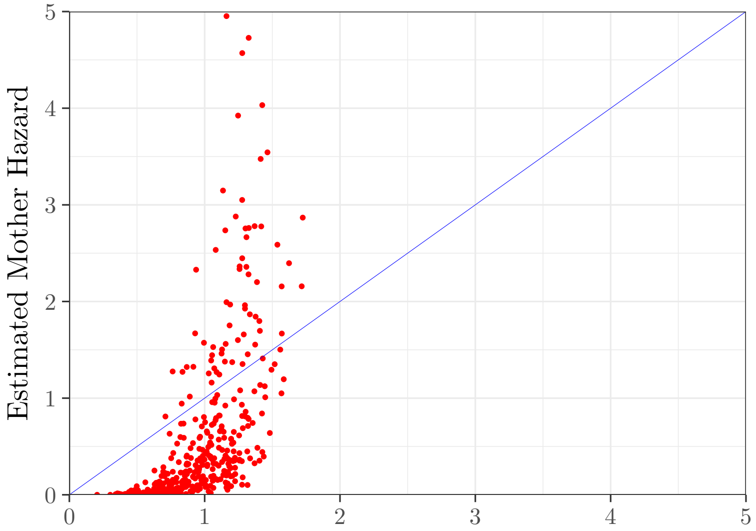


**(a)** All Ages

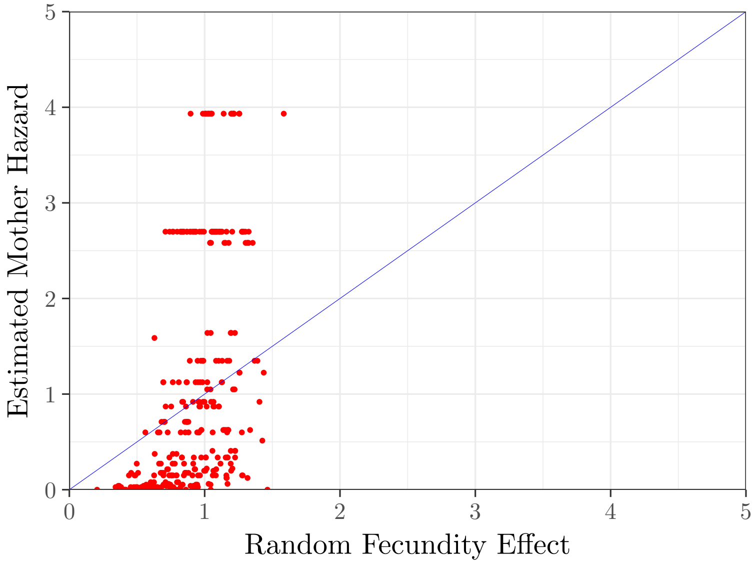


**(b)** Ages 30-4

**Figure A2** Programmed Versus Estimated Mother Fecundity, Simulation

**Table A2** An Alternative CKW Table 5

| | (1)<br>30-4 | (2)<br>30-3 | (3)<br>30-8 | (4)<br>30-4 | (5)<br>30-4 |
|---|---|---|---|---|---|
| Real wage (std) | 1.098 | 0.910 | 1.217*** | 1.071 | 1.151 |
| | (0.121) | (0.172) | (0.065) | (0.051) | (0.127) |
| Dummy for net parity 2 | 0.319*** | 0.220*** | 0.530*** | 0.396*** | 0.174*** |
| | (0.037) | (0.041) | (0.034) | (0.031) | (0.034) |
| Dummy for net parity 3 | 0.151*** | 0.060*** | 0.380*** | 0.229*** | 0.044*** |
| | (0.031) | (0.022) | (0.038) | (0.024) | (0.017) |
| Dummy for net parity 4 | 0.055*** | 0.020*** | 0.249*** | 0.135*** | 0.009*** |
| | (0.022) | (0.014) | (0.043) | (0.017) | (0.006) |
| Dummy for net parity 5 | | | | 0.068*** | |
| | | | | (0.010) | |
| Dummy for Net Parity 6 | | | | 0.045*** | |
| | | | | (0.008) | |
| Dummy for Net Parity 7 | | | | 0.028*** | |
| | | | | (0.007) | |
| Dummy for Net Parity 8+ | | | | 0.015*** | |
| | | | | (0.005) | |
| Indicator for died child | 3.479*** | 5.837*** | 2.915*** | 3.671*** | 4.754*** |
| | (0.481) | (1.218) | (0.239) | (0.240) | (0.801) |
| Indicator for last spacing interval | 0.745** | 0.618** | 0.739*** | 0.537*** | 0.598*** |
| | (0.103) | (0.142) | (0.051) | (0.045) | (0.089) |
| D31 | | | | | 1.861*** |
| | | | | | (0.287) |
| D32 | | | | | 1.800*** |
| | | | | | (0.337) |
| D33 | | | | | 3.276*** |
| | | | | | (0.881) |
| Observations | 16,187 | 12,617 | 26,792 | 43,631 | 16,187 |

*** p<0.01, ** p<0.05, * p<0.1
